# The Relationship Between Storey of Buildings and Fall Risk

**DOI:** 10.3389/fpubh.2021.665985

**Published:** 2021-11-04

**Authors:** Ching-Yao Tsai, En-Sheng Lin, Yang-Tzu Li, Tao-Hsin Tung, Wei-Cheng Chen

**Affiliations:** ^1^Department of Ophthalmology, Taipei City Hospital, Taipei, Taiwan; ^2^Institute of Public Health, National Yang-Ming Chiao-Tung University, Taipei, Taiwan; ^3^MS Program in Transdisciplinary Long Term Care and Department of Business Administration, Fu Jen Catholic University, New Taipei City, Taiwan; ^4^Department of Psychiatry, Hsinchu MacKay Memorial Hospital, Hsinchu, Taiwan; ^5^Department of Long Term Care, National Taipei University of Nursing and Health Science, Taipei, Taiwan; ^6^Taiwan Association of Health Industry Management and Development, Taipei, Taiwan; ^7^TAIWAN STIPENDIARY CO., LTD., Kaohsiung, Taiwan; ^8^Institute of Health Policy and Management, National Taiwan University, Taipei, Taiwan

**Keywords:** fall, fear of fall (FOF), risk factors, older adults, storey of building

## Abstract

**Purpose:** This study examined the association between storey of building and fall risk in older adults' residences and residents' level of fear of falling.

**Methods:** The National Health and Ageing Trends Study (NHATS) collected information that would provide an understanding of basic trends people aged 65 years and older. Using a longitudinal survey, the present study employed the first round of NHATS data that was collected in 2011. In the first round, 12,411 participants were enrolled, and 8,077 interviews were completed. The study sample sizes for falling and worry about falling are 6,153 and 6,142, respectively.

**Results:** Unadjusted analysis revealed that storey of building was a risk factor for fall and worry about falling. There was a higher prevalence for fall and worry about falling when subjects lived in single storey of building compared with the subjects live in multi-storey. Logistic regression analysis showed no highly significant between storey of building and the fall/fear of falling.

**Conclusion:** Several clinical factors independently were indicated pertaining to the fall and worry about falling in older adult's residences.

## Keypoints

- The finding of this paper indicated that the storey of building may be a risk factor for falling and develop worry about falling among elderly.
- Why does this paper matter? The results of this study could be a suggestion when arranging the living environment of elderly.

## Introduction

Falls are a major public health concern in the older adult population. For elderly people who have fallen, the fear of more falls can lead to a vicious cycle as well as result in various health problems. Millions of older people aged 65 years or older fall every year, and such incidents can cause fatal and non-fatal injuries. According to research conducted by the Centres of Disease Control and Prevention (CDC), 2.8 million older people were treated in emergency departments in the United States (US) for fall injuries in 2014. In addition, more than 27,000 older adults died from falls. In addition, annual fall injury-related costs were approximately US$31 billion (1).

Fall risk is a characteristic or a situation that is more likely to induce a fall event than other factors (2). Fall risk could be differentiated into three categories: intrinsic factors (including history of falls, age, gender, living alone, ethnicity, medicines, impaired mobility and gait, psychological factors, nutritional factors, impaired cognition, visual impairments, and sedentary behaviour), extrinsic factors (including environmental factors, footwear and clothing, and inappropriate walking aids or assistive devices), and exposure to the risk of falling (3). Some studies have considered certain hazards that increase the risk of falling, such as tripping hazards, clutter, poor lighting, low or high cabinets, no grab bar installed in the toilets, and seating that is too low or soft (4–6). Based our limited knowledge there is no study has focused on the relationship between the storey of a building that elderly people live on and fall risk. The hypothesis of this study is that elderly who lived in multi-storey of building are more likely to experience falling or report fear of falling than those who lived in single floor. Understanding this association could be essential for fall avoidance interventions. Therefore, the present study examined the association between storey of buildings and fall risk in older adults' residences and residents' level of fear of falling.

## Methods

### Study Sample

In the United States, the National Health and Ageing Trends Study (NHATS) collected information that would provide an understanding of basic trends people aged 65 years and older. Trained personnel collected the data including gender, education level, race, living environment, marital status, and economic level, by interviewing elderly people enrolled in Medicare. Using a longitudinal survey, the present study employed the first round of NHATS data that was collected in 2011. There were 8,077 interviews were completed the survey in the first round. The study excluded missing data including those who do not know answers, skipping the questions and information not ascertained. The sample sizes for falling and worry about falling are 6,153 and 6,142, respectively.

The NHATS is a publicly available data set accessed by registering online (access http://www.nhats.org) and downloading the data files for research proposes. Additional demographic data that were viewed as sensitive, such as age, were available through a simple application process. The University of Washington Human Subjects Division established that the current study did not meet the definition of research concerning human participants because the data were identified. In this study, all procedures were performed in accordance with the guidelines of our institutional ethics committee and adhered to the tenets of the Declaration of Helsinki. All patient information was anonymous. By using numerical codes for questionnaires and destroying the data in the study, the anonymity of participants and confidentiality of the responses was ensured.

### Measurements

This study examined the association between the storey of a building that elderly people live on and their fall risk. The question that “How many levels or floors are in your home?” were used to understand the storey of a building that participants lived. Meanwhile, there were several answers could be choose, including one, two, three, and more than fours. This study refined the data that elderly who live in single-floor of building when the answer was one; besides, the answer were others represented multi-storey building. The items that represented participants living in a multi-storey building included multi-unit building and other. In addition, although the NHATS did not present where falls occurred, falls defined as “any fall, slip, or trip in which you lose your balance and land on the storey or ground or at a lower level.” This study surveyed the fall or not in the past month and whether participants had worried about falling in the past month; two possible answers could be given for both items, namely yes or no.

The database in this study was used to examine the relationship between people living in single or multi-storey building vs. falling as well as the worry about falling. There were plenty of risk factors have been recognised in other studies (2–13). After refer to those studies, we conducted various independent variables are considered as potential associated factors such as gender, age, living arrangement, had knee surgery and various health condition for falling or worried about falling. Female elderly and people who were getting were more likely experienced falling and reported fear of falling. Besides, health conditions were also associated with falling or worried about falling.

### Data Analysis

All the non-response and inapplicable data are replaced in missing value. SPSS 22.0 was used for data management and to conduct statistical analysis. Chi-square (χ^2^-test) and an unadjusted odds ratio (OR) were estimated and 95% confidence interval (CI) was used to compare the relationship between storey residence, fall or not, and level of worry about falling in the past month in participants who chose to live on the single storey vs. those who chose to live on multi-storey. Multiple logistic regression was also performed in order to investigate the independence of risk factors associated with the falling or worry about falling. A *p*-value of <0.05 was considered to represent a statistically significant difference among test populations.

## Results

On one hand, the overall prevalence of fall was 10.6% (652/6,153) last month. Subjects lived in single storey of building (342/2,983 = 11.5%) had a higher prevalence for fall compared with the subjects live in multi-storey (1: 194/1,850 = 10.5%; 2: 97/1,129 = 8.6%; 3 or more: 19/191 = 9.9%). There were no statistically significant storey differences for fall among elderly (*p*-value = 0.06).

On the other hand, although there was no storey difference (*p* = 0.07) for the prevalence of worrying about the fall, elder subjects lived in single storey of building had a relative higher probability (30.1%, 896/2,980) of worrying about falling than multi-storey (1: 495/1,845 = 26.8%; 2: 310/1,126 = 27.5%; 3 or more: 51/191 = 26.7%).

Table 1 presents the unadjusted odds ratios for the association between certain relevant associated risk factors and the prevalence of fall. Compared to individuals without fall, in addition to storey (multiple vs. singles, OR = 0.83 [95%CI: 0.71–0.98]) and age (70–74 years vs. 65–69, OR = 1.83 [95%CI: 1.41–2.36], 75–79 vs. 65–69, OR = 2.10 [95%CI: 1.62–2.72], 80–84 vs. 65–69, OR = 1.65 [95%CI: 1.29–2.11], 85+ vs. 65–69, OR = 1.23 [95%CI: 0.97–1.54]), subjects featuring fall revealed a more-pronounced prevalence of: higher heart attack (yes vs. no, OR = 1.52 [95%CI: 1.23–1.86]), higher heart disease (yes vs. no, OR = 1.65, 95%CI: 1.36–1.99), higher blood pressure (yes vs. no, OR = 1.22 [95%CI: 1.02–1.46]), higher arthritis (yes vs. no, OR = 2.00 [95%CI: 1.68–2.39]), higher osteoporosis (yes vs. no, OR = 1.32 [95%CI: 1.09–1.60]), higher diabetes mellitus (yes vs. no, OR = 1.54 [95%CI: 1.29–1.83]), higher lung disease (yes vs. no, OR = 1.69 [95%CI: 1.38–2.07]), higher stroke (yes vs. no, OR = 2.10 [95%CI: 1.70–2.59]), higher dementia (yes vs. no, OR = 2.94 [95%CI: 2.28–3.80]), and higher knee surgery (yes vs. no OR = 1.53 [95%CI: 1.25–1.88]).

**Table 1:** Univariate analysis on the factors associated with fall in the past month (*n* = 6,153).

		**Fall**		**Unadjusted OR (95%CI)**	***p*-value**
		**Yes (*n* = 652)**	**No (*n* = 5,501)**		
		***N* (%)**	***N* (%)**		
Storey	Single	342(11.5)	2,641(88.5)	1.00	
	Multiple	310(9.8)	2,860(90.2)	0.83 (0.71–0.98)	0.03
Gender	Male	269(10.5)	2,292(89.5)	1.00	
	Female	343(10.5)	2,928(89.5)	1.00 (0.84–1.18)	0.98
Age (years)	65–69	103(8.7)	1,079(91.3)	1.00	
	70–74	98(7.7)	1,183(92.3)	1.83 (1.41–2.36)	<0.001
	75–79	121(9.5)	1,147(90.5)	2.10 (1.62–2.72)	
	80–84	152(12.4)	1,070(87.6)	1.65 (1.29–2.11)	
	85+	178(14.8)	1,022(85.2)	1.23 (0.97–1.54)	
Heart attack	No	131(14.4)	781(85.6)	1.00	
	Yes	520(9.9)	4,716(90.1)	1.52 (1.23–1.86)	<0.001
Heart disease	No	168(15.0)	954(85.0)	1.00	
	Yes	483(9.6)	4,534(90.4)	1.65 (1.36–1.99)	<0.001
High blood pressure	No	464(11.2)	3,684(88.8)	1.00	
	Yes	187(9.4)	1,813(90.7)	1.22 (1.02–1.46)	0.03
Arthritis	No	453(13.4)	2,916(86.6)	1.00	
	Yes	199(7.2)	2,573(92.8)	2.00 (1.68–2.39)	<0.001
Osteoporosis	No	157(12.9)	1,062(87.1)	1.00	
	Yes	495(10.1)	4,422(89.9)	1.32 (1.09–1.60)	0.004
Diabetes mellitus	No	213(13.9)	1,316(86.1)	1.00	
	Yes	439(9.5)	4,623(90.5)	1.54 (1.29–1.83)	<0.001
Lung disease	No	136(15.5)	742(84.5)	1.00	
	Yes	515(9.8)	4,755(90.2)	1.69 (1.38–2.07)	<0.001
Stroke	No	128(18.3)	573(81.7)	1.00	
	Yes	523(9.6)	4,923(90.4)	2.10 (1.70–2.59)	<0.001
Dementia	No	87(24.1)	274(75.9)	1.00	
	Yes	563(9.7)	5,224(90.3)	2.94 (2.28–3.80)	<0.001
Cancer	No	178(11.0)	1,434(89.0)	1.00	
	Yes	474(10.4)	4,064(89.6)	1.06 (0.88–1.27)	0.50
Knee surgery	No	137(14.5)	811(85.5)	1.00	
	Yes	515(9.9)	4,688(90.1)	1.53 (1.25–1.88)	<0.001
Living alone	No	169(2.8)	1,453(23.7)	1.00	
	Yes	480(7.8)	4,028(65.7)	0.97 (0.81–1.17)	0.79
Income	$4,000–$6,000	11(9.5)	105(90.5)	1.00	0.22
	< $ 2,000	35(9.5)	334(90.5)	1.09 (0.50–2.38)	0.81
	$2,000–$4,000	13(8.3)	143(91.7)	1.09 (0.61–1.95)	0.75
	$6,000–$10,000	20(16.4)	102(83.6)	1.26 (0.60–2.63)	0.53
	≥$10,000	20(10.3)	174(89.7)	0.58 (0.30–1.14)	0.11

Table 2 indicates the unadjusted odds ratios for the association between certain relevant associated risk factors and the prevalence of worrying about fall. The significant factors related to worrying about fall included storey (multiple vs. singles, OR = 0.86 [95%CI: 0.77–0.96]), gender (female vs. male, OR = 0.57 [95%CI: 0.51–0.65]), age (70–74 years vs. 65–69, OR = 3.02 [95%CI: 2.51–3.63], 75–79 vs. 65–69, OR = 2.74 [95%CI: 2.30–3.27], 80–84 vs. 65–69, OR = 2.13 [95%CI: 1.80–2.53], 85+ vs. 65–69, OR = 1.53 [95%CI: 1.30–1.81]), heart attack (yes vs. no, OR = 1.82 [95%CI: 1.59–2.09]), heart disease (yes vs. no, OR = 1.71, 95%CI: 1.51–1.94), high blood pressure (yes vs. no, OR = 2.71 [95%CI: 2.41–3.06]), arthritis (yes vs. no, OR = 2.05 [95%CI: 1.80–2.34]), osteoporosis (yes vs. no, OR = 1.48 [95%CI: 1.31–1.68]), diabetes mellitus (yes vs. no, OR = 1.67 [95%CI: 1.44–1.94]), lung disease (yes vs. no, OR = 2.08 [95%CI: 1.77–2.45]), stroke (yes vs. no, OR = 2.42 [95%CI: 1.95–3.01]), dementia (yes vs. no, OR = 1.15 [95%CI: 1.01–1.30]), cancer (yes vs. no, OR = 1.48 [95%CI: 1.27–1.71]), and knee surgery (yes vs. no OR = 1.39 [95%CI: 1.20–1.61]).

**Table 2:** Univariate analysis on the factors associated with worry about fall (*n* = 6,142).

		**Worry about fall**		**Unadjusted OR (95%CI)**	***p*-value**
		**Yes (*N* = 1,752)**	**No (*N* = 4,390)**		
		***N* (%)**	***N* (%)**		
Storey	Single	896(30.1)	2,084(69.9)	1.00	
	Multiple	856(27.1)	2,306(72.9)	0.86 (0.77–0.96)	0.01
Gender	Male	588(22.3)	2,046(77.7)	1.00	
	Female	1,164(33.2)	2,344(66.8)	0.57 (0.51–0.65)	<0.001
Age (years)	65–69	235(19.9)	946(80.1)	1.00	<0.001
	70–74	275(21.5)	1,006(78.5)	3.02 (2.51–3.63)	
	75–79	329(26.0)	936(74.0)	2.74 (2.30–3.27)	
	80–84	400(32.8)	819(67.2)	2.13 (1.80–2.53)	
	85+	513(42.9)	683(57.1)	1.53 (1.30–1.81)	
Heart attack	No	440(39.3)	680(60.7)	1.00	
	Yes	1,309(26.1)	3,701(73.9)	1.82 (1.59–2.09)	<0.001
Heart disease	No	1,322(31.9)	2,816(68.1)	1.00	
	Yes	429(21.5)	1,570(78.5)	1.71 (1.51–1.94)	<0.001
High blood pressure	No	1,253(37.3)	2,108(62.7)	1.00	
	Yes	497(17.9)	2,273(82.1)	2.71 (2.41–3.06)	<0.001
Arthritis	No	501(41.2)	715(58.8)	1.00	
	Yes	1,247(25.4)	3,663(74.6)	2.05 (1.80–2.34)	<0.001
Osteoporosis	No	531(34.8)	994(65.2)	1.00	
	Yes	1,221(26.5)	3,395(73.5)	1.48 (1.31–1.68)	<0.001
Diabetes mellitus	No	335(38.2)	543(68.1)	1.00	
	Yes	1,415(26.9)	3,845(73.1)	1.67 (1.44–1.94)	<0.001
Lung disease	No	301(43.1)	398(56.9)	1.00	
	Yes	1,447(26.6)	3,991(73.4)	2.08 (1.77–2.45)	<0.001
Stroke	No	169(47.7)	185(53.2)	1.00	
	Yes	1,582(27.4)	4,201(72.6)	2.42 (1.95–3.01)	<0.001
Dementia	No	494(30.7)	1,115(69.3)	1.00	
	Yes	1,258(27.8)	3,272(72.2)	1.15 (1.01–1.30)	0.02
Cancer	No	325(35.7)	585(64.3)	1.00	
	Yes	1,426(27.3)	3,802(72.7)	1.48 (1.27–1.71)	<0.001
Knee surgery	No	327(34.5)	620(65.5)	1.00	
	Yes	1,425(27.4)	3,769(72.6)	1.39 (1.20–1.61)	<0.001
Living alone	No	534(29.1)	1,298(70.9)	1.00	
	Yes	1,085(28.5)	2,717(71.5)	1.03 (0.91–1.16)	0.63
Income	$4,000–$6,000	26(23.4)	85(76.6)	1.00	0.74
	< $ 2,000	94(26.9)	255(73.1)	1.30 (0.75–2.25)	0.37
	$2,000–$4,000	35(25.0)	105(75.0)	1.08 (0.72–1.61)	0.99
	$6,000–$10,000	31(30.7)	70(69.3)	1.20 (0.72–1.97)	0.57
	≥$10,000	52(28.6)	130(71.4)	0.93 (0.53–1.53)	0.36

The effect of independent associated risk factors on fall was examined using a multiple logistic regression model. As depicted in Table 3, subsequent to adjustment for confounding factors, the following appeared to be significantly related to fall prevalence: age (85+ years vs. 65–69 years, adjusted OR = 1.43 [95 CI: 1.09–1.88]), arthritis (yes vs. no, adjusted OR = 1.64 [95%CI: 1.37–1.97]), diabetes mellitus (yes vs. no, adjusted OR = 1.45 [95%CI: 1.20–1.74]), lung disease (yes vs. no, adjusted OR = 1.51 [95%CI: 1.22–1.87]), stroke (yes vs. no, adjusted OR = 1.54 [95%CI: 1.23–1.93]), dementia (yes vs. no, adjusted OR = 2.17 [95%CI: 1.64–2.85]), and knee surgery (yes vs. no, adjusted OR = 1.38 [95%CI: 1.12–1.71]).

**Table 3:** Multiple logistic regression model of fall in the past month (*n* = 6,153).

	**Fall (Yes vs. No)**					
	**β**	**SE**	***p*-value**	**OR**	**95% CI**	
					**Lower**	**Upper**
Storey (mulitple vs. single)	−0.09	0.85	0.25	0.90	0.76	1.07
**Age (years)**						
70–74 vs. 65–69	−0.22	0.15	0.14	0.80	0.59	1.07
75–79 vs. 65–69	−0.07	0.14	0.61	0.92	0.69	1.23
80–84 vs. 65–69	0.22	0.13	0.10	1.25	0.95	1.64
85+ vs. 65–69	0.36	0.13	0.01	1.43	1.09	1.88
Heart attack (yes vs. no)	0.11	0.11	0.32	1.12	0.89	1.41
Heart disease (yes vs. no)	0.19	0.10	0.06	1.21	0.98	1.50
High blood pressure (yes vs. no)	−0.04	0.09	0.63	0.95	0.79	1.15
Arthritis (yes vs. no)	0.49	0.09	<0.001	1.64	1.37	1.97
Diabetes mellitus (yes vs. no)	0.37	0.09	<0.001	1.45	1.20	1.74
Lung disease (yes vs. no)	0.41	0.10	<0.001	1.51	1.22	1.87
Stroke (yes vs. no)	0.43	0.11	<0.001	1.54	1.23	1.93
Dementia (yes vs. no)	0.77	0.14	<0.001	2.17	1.64	2.85
Knee surgery (yes vs. no)	0.32	0.10	0.002	1.38	1.12	1.71

The effects of the independent associated factors of worry about fall were also examined by the multiple logistic regression model (Table 4). Independent factors of worry about fall included gender (female vs. male, adjusted OR = 0.71 [95%CI: 0.62–0.81]), age (80–84 years vs. 65–69 years, adjusted OR = 1.65 [95 CI: 1.35–2.01], 85+ years vs. 65–69 years, adjusted OR = 2.48 [95 CI: 2.03–3.02]), heart attack (yes vs. no, adjusted OR = 1.36 [95%CI: 1.16–1.58]), heart disease (yes vs. no, adjusted OR = 1.35 [95%CI: 1.18–1.55]), high blood pressure (yes vs. no, adjusted OR = 2.10 [95%CI: 1.85–2.39]), arthritis (yes vs. no, adjusted OR = 1.51 [95%CI: 1.30–1.75]), osteoporosis (yes vs. no, adjusted OR = 1.40 [95%CI: 1.22–1.61]), diabetes mellitus (yes vs. no, adjusted OR = 1.41 [95%CI: 1.20–1.65]), lung disease (yes vs. no, adjusted OR = 1.53 [95%CI: 1.28–1.83]), stroke (yes vs. no, adjusted OR = 1.45 [95%CI: 1.15–1.84]), and knee surgery (yes vs. no, adjusted OR = 1.20 [95%CI: 1.02–1.41]) after adjustment for confounding factors.

**Table 4:** Multiple logistic regression model of worry about fall (*n* = 6,142).

	**Worry about fall (Yes vs. No)**					
	**β**	**SE**	***p*-value**	**OR**	**95% CI**	
					**Lower**	**Upper**
Storey (mulitple vs. single)	−0.016	0.061	0.79	0.98	0.87	1.10
Gender (female vs. male)	−0.33	0.06	<0.001	0.71	0.62	0.81
**Age (years)**						
70–74 vs. 65–69	0.00	0.10	0.97	1.00	0.81	1.23
75–79 vs. 65–69	0.17	0.10	0.08	1.18	0.97	1.44
80–84 vs. 65–69	0.50	0.10	<0.001	1.65	1.35	2.01
85+ vs. 65–69	0.90	0.10	<0.001	2.48	2.03	3.02
Heart attack (yes vs. no)	0.30	0.07	<0.001	1.36	1.16	1.58
Heart disease (yes vs. no)	0.30	0.06	<0.001	1.35	1.18	1.55
High blood pressure (yes vs. no)	0.74	0.06	<0.001	2.10	1.85	2.39
Arthritis (yes vs. no)	0.41	0.07	<0.001	1.51	1.30	1.75
Osteoporosis (yes vs. no)	0.34	0.07	<0.001	1.40	1.22	1.61
Diabetes mellitus (yes vs. no)	0.34	0.08	<0.001	1.41	1.20	1.65
Lung disease (yes vs. no)	0.42	0.09	0.002	1.53	1.28	1.83
Stroke (yes vs. no)	0.37	0.12	0.24	1.45	1.15	1.84
Dementia (yes vs. no)	0.08	0.06	0.53	1.08	0.94	1.24
Cancer (yes vs. no)	0.05	0.08	0.42	1.05	0.89	1.25
Knee surgery (yes vs. no)	0.18	0.08	<0.001	1.20	1.02	1.41

## Discussion

### Clinical Implications

Falls are the leading cause of injury in the elderly population. A serious fall could result in decreased independent function and quality of life. Hip fractures in particular are a serious consequence of falling that could be devastating in older subjects. Table 5 presents the outcome evaluation of fall in older adults' residences in various populations (7–14). In this study, the results revealed that older adults who lived in multi-storey of buildings were not more worried about falling than lived in single-storey. Although the reason why older adults would not worry about the fall when they living in more than one storey of building was not clear, we supposed that disabled elderly who were inclined to experience falling or reported fear of falling were more likely to live in one storey of building. On the other hand, the barriers and safety conditions were difference between single-storey and multi-storey houses.

**Table 5:** The outcome evaluation of fall in older adults' residences in various populations.

**First author**	**Study year**	**Screened number**	**Following period**	**Setting**	**Outcome**	**Risk estimate**	**Reference**
Coutinho ES	2012	250 patients aged 60 years and over	1 year	Brasil	The one-year cumulative mortality was 25.2% in the case of individuals with severe fractures and 4% for those individuals without	NA	(8)
Gilasi HR	2015	424 elderly people	2010–2012	Qom, Iran	40 elderly persons (9.4%) due to falls died in the hospital	NA	(9)
Stewart BT	2016	5,148 individuals.	2003–2014	Baghdad, Iraq	5 persons died as a result of falling.	Respondents who spent significant time within the home had three times greater odds of having suffered a fall injury than student referents (OR 3.34; 95%CI 1.30–8.60).	(10)
Deprey SM	2017	389,891 Waukesha county population from the 2010 census	2005–2012	Wisconsin, United States	842 fall-related deaths were identified in Waukesha county from 2005–2012	Advancing age (OR = 1.05,95% CI = 1.02–1.08)	(11)
Daoust R	2018	67,929 patients for the final sample	2004–2014.	Quebec, Canada	4.02% Predictors associated with death in hospital for patients with fall as the injury mechanism	Patients who filled an opioid prescription within 2 weeks before falling were at increased risk (OR = 1.59; 95% CI 1.35–1.87)	(12)
Zhang L	2019	260 participants aged 60+ years	12-month	Xiamen, China	15.9% of residents suffered a hip fracture and died within a month from complications.	Between ADL and feet and footwear (OR = 3.120, *P* < .001; OR = 3.010, *p* = 0.007 in Models 1 and 3)Between ADL and cognitive status (OR = 4.401, *P* < 0.001; OR = 4.101, *p* = 0.005 in Models 2 and 3)	(13)
Oh J	2019	4,386 subjects aged 50 years and over	7.8 years	Korean	Number of death: Men: 255 Woman: 146	Subjects who were moderately or very afraid of falling had a higher mortality rate (HR: 1.26, 95% CI: 0.97–1.63)	(14)
Barker A	2019	430 people aged 60–90 years	Between 1 April 2014 and 29 June 2015	Australia	There were 2 in the RESPOND group and 1 in the control group.	NA	(15)

From the clinical viewpoint, previous studies reported that physical inactivity can increase depressive symptoms and a significant association between physical inactivity and major non-communicable diseases, such as coronary heart disease, type 2 diabetes, breast cancers and colon cancers, which can also shorten life expectancy (15, 16). There were several risk factors for falls in older adults has been recognised (17–25). However, no study has assessed the relationship between the storey on which elderly people live on and other fall-related variables such as fall risks and level of worry about falls. The results of this study revealed trend between storey number and falling, but it was not highly significant (*p*-value = 0.06). In addition, the association between storey number and participants' level of worry about falling also was not significant (*p*-value = 0.07). The results indicated that no significant relationship between the storey of a building, the risk of falling, and level of worry about fall compared with other related factors. Elderly peoples' physical ability could become increasingly restricted. In this study, we did not prove our hypothesis that elderly people who lived on higher storey worry more about falling because of the fear of going up and down stairs.

### Methodological Considerations

Although this study provided new insights into the relationship between the storey number of a building and level of worry about falling in elderly people, it still had several limitations. This study evaluated an extensive array of fall risk factors, however, some restrictions existed in the NHATS investigation, such as the lack of data on environmental factors, footwear and clothing, inappropriate walking aids or assistive devices, and gait. Secondly, the misclassification bias of on floor number from 0 to 3, etc. could be occurred. In this study, to get classified as living in a multi-storey building, it is clear to form this that participants would be classified as multi-storey, even if they lived on the ground floor of a multi-storey building. While one possibility for being classified as single-storey living was that participants lived in a “free-standing single house”: this mean it would have to be a bungalow with only one storey, or could this term include two storey houses with bedrooms upstairs, a standard house in the UK. If single storey does include two storey houses, then some of the participants in the single storey group would have to climb stairs to get to bed. Hence, the results detailed may differ if applied to specific floors. Thirdly, the missing response of fall or worry about fall are not only low it strengthens study results, but also if the numbers drop off considerably it is an indication of potential selection bias. Fourthly, no information about stair lifts does indicate a another weakness of the study, as whether or not a multi-storey home has an elevator, is clearly important in relation to fall risk and fear of fall in the home and the lack of this information may explain why we failed to find an association. Fifthly, the data from NHATS were secondary that may deduce the link between each risk factor, including the storey of building. Besides, this was a cross-sectional study, which limited its ability to draw causal inferences. To conduct a number of prospective longitudinal analogous studies could solute such a quandary would best be accomplished. The results of which would be also expected to complement the cross-sectional findings of this study. However, one key variable that could be collected over time is falling. In a different type of study, they could have asked participants to complete falls diaries. It is not clear whether residence is likely to change greatly over time, that is their status in relation to the floor they live on might not change. A change in residence (to institutional care) would for many people indicate a deterioration in the condition, and this might be picked up as a move from a house to multi-storey accommodation, which could confound a floor of residence analysis.

## Conclusion

Several clinical factors independently were indicated pertaining to the falling and worry about falling in older adult's residences. Further studies not only are needed to evaluate the temporal sequence of events that typically lead to falling, but also studies should assess how gender-related differences are related of falling in older adult's residences.

## Data Availability Statement

The datasets presented in this study can be found in online repositories. The names of the repository/repositories and accession number(s) can be found below: https://nhats.org/researcher/nhats.

## Author Contributions

C-YT, E-SL, T-HT, and W-CC conducted the study and drafted the manuscript. E-SL, Y-TL, T-HT, and W-CC participated in the design of the study and performed data analysis. C-YT, T-HT, and W-CC conceived the study and participated in its design and coordination. All of the authors read and approved the final manuscript.

## Conflict of Interest

The authors declare that the research was conducted in the absence of any commercial or financial relationships that could be construed as a potential conflict of interest.

## Publisher's Note

All claims expressed in this article are solely those of the authors and do not necessarily represent those of their affiliated organizations, or those of the publisher, the editors and the reviewers. Any product that may be evaluated in this article, or claim that may be made by its manufacturer, is not guaranteed or endorsed by the publisher.
